# The B7H4-PDL1 classifier stratifies immuno-phenotype in cervical cancer

**DOI:** 10.1186/s12935-021-02423-8

**Published:** 2022-01-04

**Authors:** Lingyan Chen, Jianfeng Dong, Zeying Li, Yu Chen, Yan Zhang

**Affiliations:** 1 Department of Oncology, Wuxi Maternal and Child Health Hospital Affiliated to Nanjing Medical University, No. 48 Huaishu Road, Wuxi, 214000 China; 2 Department of Pathology, Wuxi Maternal and Child Health Hospital Affiliated to Nanjing Medical University, Wuxi, 214000 China; 3 Wuxi Clinical Medical College, Nanjing Medical University, Wuxi, 214000 China

**Keywords:** B7H4, PDL1, Tumor immunity, Pan-cancer

## Abstract

**Background:**

It has been revealed that B7H4 is negatively correlated with PDL1 and identifies immuno-cold tumors in glioma. However, the application of the B7H4-PDL1 classifier in cancers has not been well testified.

**Methods:**

A pan-cancer analysis was conducted to evaluate the immunological role of B7H4 using the RNA-sequencing data downloaded from the Cancer Genome Atlas (TCGA). Immunohistochemistry (IHC) and multiplexed quantitative immunofluorescence (QIF) were performed to validate the primary results revealed by bioinformatics analysis.

**Results:**

The pan-cancer analysis revealed that B7H4 was negatively correlated with PDL1 expression and immune cell infiltration in CeCa. In addition, patients with high B7H4 exhibited the shortest overall survival (OS) and relapse-free survival (RFS) while those with high PDL1 exhibited a better prognosis. Multiplexed QIF showed that B7H4 was mutually exclusive with PDL1 expression and the B7H4-high group exhibited the lowest CD8 + T cell infiltration. Besides, B7H4-high predicted highly proliferative subtypes, which expressed the highest Ki67 antigen. Moreover, B7H4-high also indicated a lower response to multiple therapies.

**Conclusions:**

Totally, the B7H4-PDL1 classifier identifies the immunogenicity and predicts proliferative subtypes and limited therapeutic options in CeCa, which may be a convenient and feasible biomarker in clinical practice.

**Supplementary Information:**

The online version contains supplementary material available at 10.1186/s12935-021-02423-8.

## Background

With the development of treatment strategies for malignant cancers, immune checkpoint blockade (ICB) is emerging as inspiring immunotherapy transforming the standard of treatment [1]. Representative immune checkpoint inhibitors are monoclonal antibodies that interfere with the interaction between programmed cell death 1 (PD1) and programmed death-ligand 1 (PDL1) inhibitory proteins expressed on the surface of T cells and tumor cells, respectively [2, 3]. High infiltrating levels of effector immune cells are essential for the response to immunotherapy. Typically, tumors with a high effector immune cells infiltration are defined as “hot” tumors, in which PDL1 is adaptively upregulated [4]. In clinical practice, the detection of PDL1 is considered a predictive biomarker for anti-PD1/PDL1 therapies [5]. Patients with high PDL1 expression tended to be highly responsive to PD1/PDL1 blockade [6].

In addition to PDL1, B7H4 is highly expressed in tumor tissues, making them attractive candidate immunotherapeutic targets and biomarkers [7, 8]. B7H4 was first discovered in 2003 due to its sequence similarities with other B7 family members [9, 10]. B7H4 encodes a heavily glycosylated membrane protein, and negatively regulates T cell activation by limiting proliferation, cytokine production, and cytotoxicity [9, 10]. According to previous research, co-expression of PDL1 and B7H4 is rare in several cancers, including lung cancer and breast cancer [11, 12]. Moreover, B7H4 is negatively correlated with PDL1 and identifies immuno-cold tumors in glioma [13]. However, as we all know, the blood–brain barrier blocks the entry of the immune cells and therapeutic drugs to tumor tissues and brain parenchyma to a great extent [14]. Thus, the application of the B7H4-PDL1 classifier in glioma might not be a good option.

In the current research, we conducted a pan-cancer analysis of the correlation between B7H4 and immunological features. We found that B7H4 was negatively correlated with immunological features in several cancers, including cervical cancer (CeCa), glioma and melanoma. We selected CeCa for further analysis and found that B7H4 was negatively correlated with PDL1 and immune cells infiltration. Briefly, B7H4 high expression identifies immuno-cold subtype in CeCa. It was also suggested that B7H4 predicted the hyperproliferative subtype in CeCa. Overall, the application of the B7H4-PDL1 classifier in CeCa may be a better choice.

## Methods

### Public data acquisition and Bioinformatics analysis

The pan-cancer normalized RNA-sequencing (RNA-seq) datasets and corresponding clinical information from the Cancer Genome Atlas (TCGA) dataset were downloaded from Xena (https://xenabrowser.net/datapages/). The abbreviations for various cancer types were given in Additional file 1: Table S1. Besides, the data of RNA-seq and clinical information in the Chinese Glioma Genome Atlas (CGGA) database (mRNA-array_301) were also obtained from the official website (http://www.cgga.org.cn/index.jsp).

The bioinformatics analysis in this research contained public data acquisition, pan-cancer analysis of the correlation between B7H4 and immunological features, evaluation of the immunological features of the TME in CeCa, calculation of the enrichment scores of immunotherapy-related gene signatures and prediction of therapeutic response. The detailed description of bioinformatics analysis was exhibited in Additional file 1: Additional Methods.

### Clinical samples

The CeCa tissue microarray (TMA, Cat. HUteS168Su01) was obtained from Outdo BioTech (Shanghai, China). The TMA contained 126 CeCa tissues and 42 paired adjacent tissues. Detailed clinico-pathological characteristics follow-up data of the cohorts were provided by Outdo BioTech as well and shown in original data. Besides, we also 30 CeCa tissues submitted for multiplexed quantitative immunofluorescence (QIF) in this research and all the patients were recruited by the Wuxi Maternal and Child Health Hospital Affiliated to Nanjing Medical University. Ethical approval for the use of the TMA was granted by the Clinical Research Ethics Committee in Wuxi Maternal and Child Health Hospital Affiliated to Nanjing Medical University.

### IHC and semi-quantitative evaluation

Immunohistochemistry (IHC) staining was conducted on the TMA. The primary antibodies used were as follows: anti-B7H4 (1:100 dilution, Cat. ab252438, Abcam, Cambridge, UK), anti-PD-L1 (Ready-to-use, Cat. GT2280, GeneTech, Shanghai, China), anti-KI67 (Ready-to-use, Cat. GT2101, GeneTech, Shanghai, China) and anti-EGFR (Ready-to-use, Cat. PA192, Abcarta, Suzhou, China). Antibody staining was visualized with DAB and hematoxylin counterstain, and stained sections were scanned using Aperio Digital Pathology Slide Scanners. A total of 118 TMA points were retained for further analysis after the exfoliated points were removed. All stained sections were independently evaluated by two independent pathologists using the immunoreactivity score (IRS) criterion [15].

### Definition of the positive rates of B7H4 and PDL1 in CeCa

To identify the immune subtypes in CeCa based on the B7H4-PDL1 classifier, we first defined the positive staining with the threshold of IRS ≥ 3 in the IHC cohort. Thus, PDL1 was 36.44% positive in the cohort and B7H4 was 19.49%. For the patients in the TCGA dataset, we defined PDL1 as positive expression with the threshold of top 35% and B7H4 as positive expression with the threshold of top 20% by referring to positive rate in the IHC cohort.

### Multiplexed quantitative immunofluorescence

To measure the levels of PD-L1, B7-H4 and CD8 in the CeCa samples, the multiplexed QIF was directly performed on the tissue section using a previously described protocol with simultaneous detection of DAPI [16]. The primary antibodies were as follows: anti-B7H4 (1:100 dilution, Cat. ab252438, Abcam, Cambridge, UK), anti-PDL1 (1:500 dilution, Cat. ab237726, Abcam, Cambridge, UK) and anti-CD8 (1:200 dilution, Cat. ab101500, Abcam, Cambridge, UK). The expression levels of B7H4 and PDL1 were evaluated according to the previous method. For CD8 staining, infiltration level was assessed by estimating the percentage of cells with strong intensity of membrane staining in the stroma cells. For stratification, the B7H4 and PDL1 levels were classified as high/low using the top 50-percentile of the cohort scores as stratification cut-point.

### Statistical analysis

All statistical analyses were applied by R version 4.0.0. Wilcoxon rank-sum test was used to measure the difference between groups with continuous values, while Fisher exact method for evaluating the difference among grouping variables. For all analyses, a two-paired P-value < 0.05 was regarded as statistical significance if not noted.

## Results

### The prognostic value of the B7H4-PDL1 classifier in glioma

**Fig. 1:**
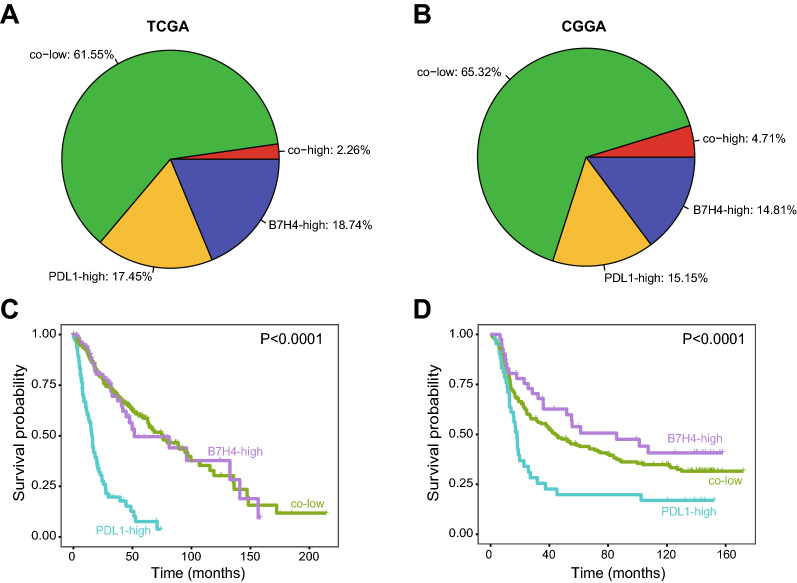
The prognostic value of the B7H4-PDL1 classifier in glioma. **A**, **B** The proportion of B7H4-high, PDL1-high, co-low and co-high subgroups in glioma in TCGA and CGGA dataset. **C**, **D** The prognostic value of the B7H4-PDL1 classifier in glioma in TCGA and CGGA datasets

According to the report of Chen et al*.*, the positive rates of B7H4 and PDL1 were approximately 20% revealed by IHC analysis [13]. We defined the positive B7H4 and PDL1 expression as the top 20% expression, and then divide glioma patients into three major subgroups in both TCGA and CGGA datasets. Similarly, the co-high expression of B7H4 and PDL1 was rare (Fig. 1A, B). Besides, Chen et al*.* declared that the B7H4-PDL1 classifier distinguished different immunogenicity in glioma [13]. Based on molecular characteristics, B7H4-high tumors could be defined as “cold” tumors, PDL1-high tumors were “hot” tumors, and co-low tumors were somewhere in between. Generally, patients with “hot” tumors exhibited a better prognosis and higher therapeutic response [17–19]. However, this assumption was not supported by survival analysis. As shown in Additional file 2: Figure S1, patients with high PDL1 expression exhibited the shortest overall survival (OS) (Fig. 1C, D). What’s more, in the CGGA dataset, patients with high B7H4 expression exhibited the longest OS, but whose gliomas should be defined as “cold” tumors (Fig. 1D). Overall, these data uncover the B7H4-PDL1 classifier maybe not a better choice in glioma, especially among the Chinese population.

### Pan-cancer immunological correlation of B7H4

**Fig. 2:**
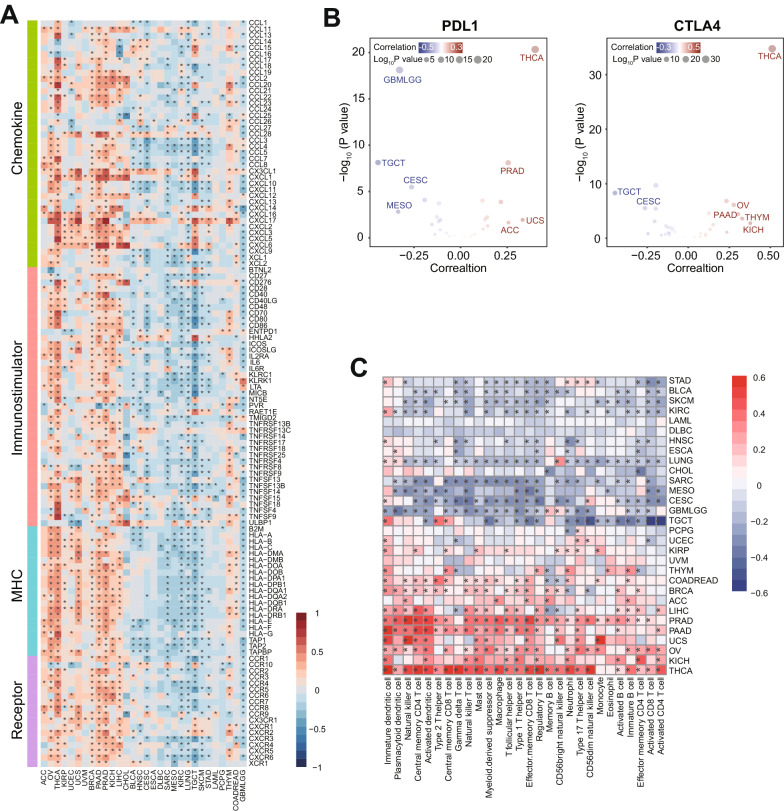
Correlations between B7H4 and immunological features in pan-cancer. **A** The correlation between B7H4 and 122 immunomodulators. The color reveals the correlation coefficient. The asterisks reveal statistical differences assessed by Pearson analysis. **B** The correlations between B7H4 and PDL1 as well as CTLA4. The dots symbolize cancer types. **C** The correlations between B7H4 and 28 TIICs calculated with the ssGSEA algorithm. The color reveals the correlation coefficient. The asterisks reveal statistical differences assessed by Pearson analysis

To explore the appropriate cancer types in which the B7H4-PDL1 classifier could be applied, we analyzed the immunological correlation of B7H4 in different types of cancers using RNA-seq datasets from the TCGA project. We found that B7H4 expression was negatively correlated with immunomodulators, immune checkpoints and TIICs infiltration in testicular germ cell tumor and CeCa (Fig. 2A–C). Thus, we chose CeCa for further analysis due to its relatively high incidence.

### B7H4 and PDL1 are mutually exclusive expression in CeCa

**Fig. 3:**
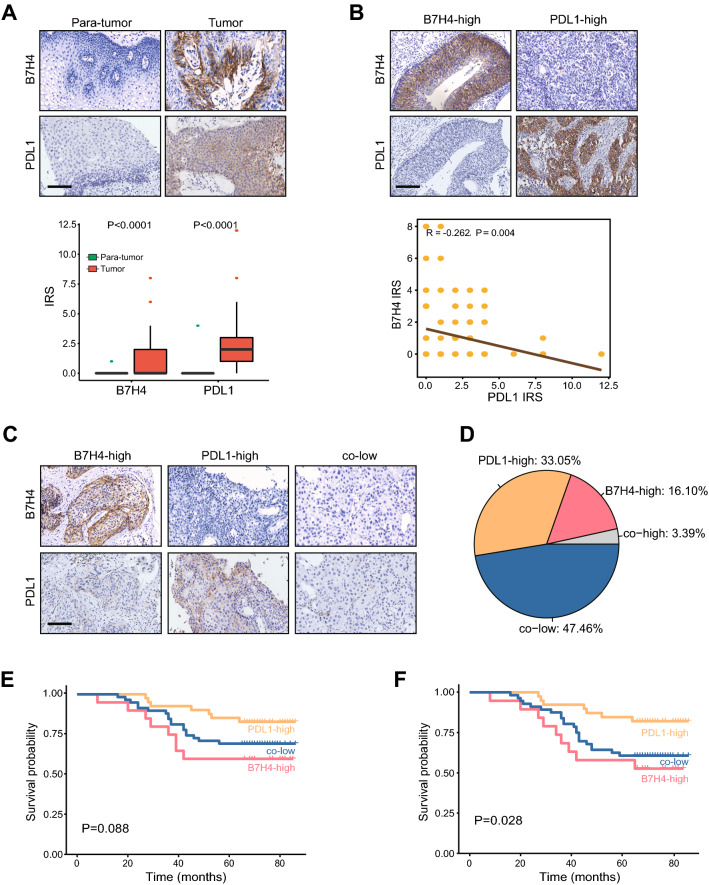
Expression of B7H4 and PDL1 and prognostic value of the B7H4-PDL1 classifier in CeCa. **A** Expression levels of B7H4 and PDL1 in CeCa and para-tumor tissues. **B** The correlation of B7H4 and PDL1 expression CeCa tissues. **C** Representative images revealing B7H4 and PDL1 expression in different subtypes using anti-B7H4 staining and anti-PDL1 staining. Magnification, 200 × , Bar = 100 μm. **D** The proportion of B7H4-high, PDL1-high, co-low and co-high subgroups in CeCa. **E**, **F** The prognostic value of the B7H4-PDL1 classifier in predicting OS and RFS in CeCa

Next, IHC analysis was performed on the CeCa TMA using the antibodies against B7H4 and PDL1. The results showed that B7H4 and PDL1 were highly expressed in the tumor tissues compared with adjacent tumors, and the positive rates for B7H4 and PDL1 were 19.49% and 36.44%, respectively (Fig. 3A). Besides, similar to the bioinformatics analysis, B7H4 expression was negatively correlated with PDL1 (Fig. 3B). T cell inflamed score is developed using IFN-γ-related mRNA profiles to predict clinical response to PD-1 blockade [20]. In the TCGA dataset, B7H4 expression was negatively while PDL1 expression positively correlated with T cell inflamed score (Additional file 2: Figure S1A, S1B). Besides, B7H4 was also negatively correlated with most immune checkpoints in addition to PDL1 and CTLA4 (Additional file 2: Figure S1C). We subsequently divide CeCa patients into three major subgroups based on B7H4 and PDL1 expression namely B7H4-high, PDL1-high and co-low groups, and the co-high group was excluded from the current analysis due to the limited proportion (Fig. 3C, D). The survival analysis was performed, and the results showed that patients with high B7H4 exhibited the shortest OS and relapse-free survival (RFS) while those with high PDL1 exhibited a better prognosis (Fig. 3E, F). Taken together, these data support that the B7H4-PDL1 classifier may be suitable in CeCa.

### B7H4-high identifies immuno-cold tumors in CeCa

**Fig. 4:**
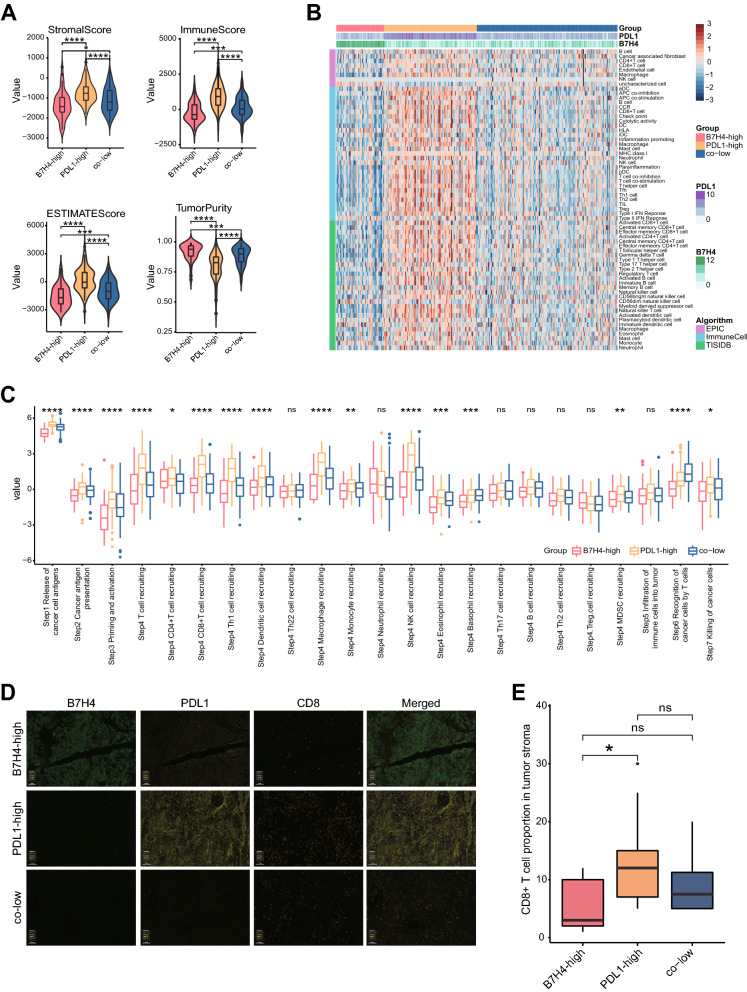
Correlations between the B7H4-PDL1 classifier and immunological features in CeCa. **A** Differences in Tumor Purity, ESTIMATE Score, Immune Score, and Stromal Score estimating by ESTIMATE algorithm in B7H4-high, PDL1-high and co-low subgroups. *P < 0.05; ***P < 0.001; ****P < 0.0001. **B** Differences in the levels of TIICs calculated using three algorithms in B7H4-high, PDL1-high and co-low subgroups. **C** Differences in the various steps of the cancer immunity cycle in B7H4-high, PDL1-high and co-low subgroups. Ns: no significant difference; *P < 0.05; **P < 0.01; ***P < 0.001; ****P < 0.0001. **D** Representative images revealing B7H4, PDL1 and CD8 expression in different subtypes revealed by multiplexed QIF. Magnification, ×200, Bar = 100 μm. **E** Differences in CD8 + T cell infiltration in B7H4-high, PDL1-high and co-low subgroups

We next evaluated whether the B7H4-PDL1 classifier was correlated with tumor immunogenicity in CeCa. For the patients in the TCGA dataset, we defined PDL1 as a positive expression with the threshold of top 35% and B7H4 as a positive expression with the threshold of top 20% by referring to a positive rate in the IHC cohort. Then, three major subgroups based on B7H4 and PDL1 expression were divided (Additional file 3: Figure S2A). As expected, according to the ESTIMATE algorithm, the B7H4-high group exhibited the lowest stromal score, immune score and ESTIMATE score, but highest tumor purity (Fig. 4A). In addition, most immune cell markers were lowly expressed in the B7H4-high group (Additional file 3: Figure S2B). We also estimated the infiltrating abundance of TIICs using three independent algorithms, and the results also supported that B7H4-high identifies immuno-cold tumors with the lowest TIICs infiltration (Fig. 4B). Besides, activities of most steps in the cycle were revealed to be lowest in the B7H4-high group (Fig. 4C). Moreover, T cell inflamed score was also lowest in the B7H4-high group (Additional file 2: Figure S2C). We also performed multiplexed QIF using the antibodies against B7H4, PDL1 and CD8 (Fig. 4D). As expected, the B7H4-high group exhibited the lowest CD8 + T cell levels and the PDL1-high group exhibited the highest CD8 + T cell levels (Fig. 4E). Collectively, these data support that B7H4-high identifies immuno-cold tumors in CeCa.

### B7H4-high identifies highly proliferative tumors and predicts limited therapeutic potions in CeCa

**Fig. 5:**
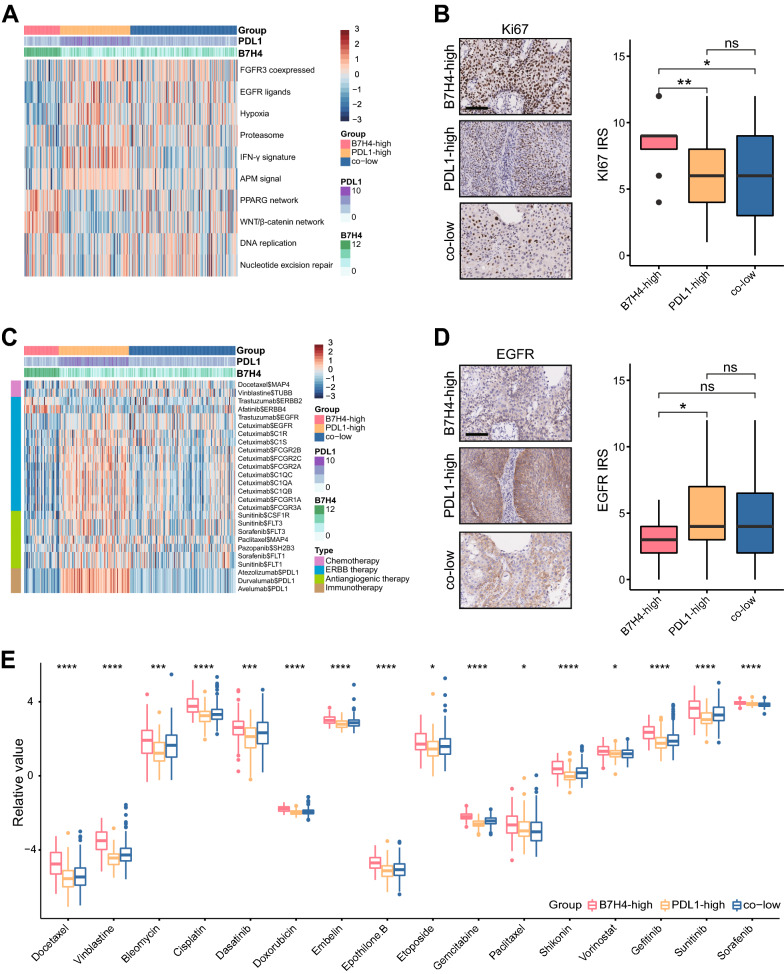
B7H4-high predicts proliferative subtype and limited therapeutic options in CeCa. **A** Differences in enrichment scores of several therapeutic signatures in B7H4-high, PDL1-high and co-low subgroups. **B** Differences in Ki67 expression B7H4-high, PDL1-high and co-low subgroups. **C** Differences in the drug-target genes expression in B7H4-high, PDL1-high and co-low subgroups. **D** Differences in EGFR expression in B7H4-high, PDL1-high and co-low subgroups. **E** Differences in IC50 of common anti-cancer drugs in B7H4-high, PDL1-high and co-low subgroups. *P < 0.05; ***P < 0.001; ****P < 0.0001

Subsequently, we evaluated whether the B7H4-PDL1 classifier was correlated with anti-tumor therapies in CeCa. As shown in Fig. 5A, several positively immune-related gene signatures exhibited the lowest scores in the B7H4-high group, the scores of PPARG and Wnt/β-catenin networks were highest in the B7H4-high group. In addition to negatively regulating anti-tumor immunity, these pathways were also correlated with high proliferation [21, 22]. Thus, Ki67 was stained by IHC analysis to compare the proliferation of tumors. We found that Ki67 was highest expressed in the B7H4-high group (Fig. 5B). Moreover, findings from the Drugbank database revealed a remarkably lowest response to chemotherapy, anti-ERBB (excluding anti-ERBB2 and anti-ERBB4) therapy, antiangiogenic therapy and immunotherapy in the B7H4-high group (Fig. 5C). Besides, IHC analysis validated that EGFR, the main target for anti-ERBB therapy, was lowest expressed in the B7H4-high group (Fig. 5D). Moreover, IC50 of anti-cancer drugs in patients from the TCGA database according to the pRRophetic algorithm was estimated. The results showed patients with high B7H4 expression were more sensitive to common anti-cancer drugs (Fig. 5E). To sum up, B7H4 is an indicator for highly proliferative subtype and limited therapeutic options.

## Discussion

Solid tumors are made up not only of tumor cells, but also of a large number of stromal cells, including immune cells, fibroblasts, etc*.*[23]. In principle, tumors could be divided into “cold” or “hot” depending on the characteristics of TME. “Cold” tumors are featured with immunosuppressive TME and resistance to either immunotherapy or chemotherapy, but “hot” tumors exhibit higher response rates to these therapies, which are characterized by T cell infiltration, increased interferon-γ (IFN-γ) signaling, activation of inhibitory checkpoints (CTLA4, PDL1, etc.), genomic instability and the activation of major histocompatibility complex class I (MHC-I) [19, 24]. Driving the formation of “hot” tumors is a gradual and complicated process. Extensive efforts have been made to convert cold tumors to hot tumors, but the clinical applications are limited [25]. However, using reliable biomarkers to distinguish “hot” and “cold” tumors is a convenient and feasible strategy.

PDL1 has been identified as an immunosuppressive molecule that negatively regulates cytotoxic immune cells [26]. However, the correlation between PDL1 expression and anti-tumor immunity remains of considerable controversy in human cancers. High PDL1 expression is a striking feature of “hot” tumors and predicts actionable immune activation [27]. Some scholars believe that it is necessary to distinguish constitutive and adaptive PDL1 expression. Constitutive PDL1 has been traditionally considered as a negative co-stimulatory molecule promoted by oncogenic driver mutations [28]. In some cases, PDL1 expression is primarily induced by activated CD8 + T cells via IFN-γ signaling and predicts pre-existing adaptive immune response [29]. Thus, the expression of adaptive PDL1 indicates higher response rates to both immunotherapy and chemotherapy.

B7H4 is another negative co-stimulatory molecule and negatively regulates T cell activation by inhibiting its activity [9, 10]. Increasing numbers of research have uncovered that B7H4 was highly expressed in tumor cells and the increased expression predicted poor prognosis in multiple cancers [30–32]. According to the report presented by Chen et al*.*, B7H4 is negatively correlated with PDL1 and identifies immuno-cold tumors in glioma [13]. However, considering that the brain is traditionally an immune-exempt organ as well as the inconsistency between the subtypes demarcated the B7H4-PDL1 classifier and prognosis [14], the application of the B7H4-PDL1 classifier is not a good option in glioma. In this research, we first conducted a pan-cancer analysis of immunological correlation of B7H4, we found that B7H4 negatively correlated with immunomodulators, immune checkpoints and TIICs infiltration in CeCa. Further validation suggested B7H4 and PDL1 were mutually exclusive expression in CeCa. In-depth analysis revealed that the levels of immune infiltration and activation were various in these three groups, and the B7H4-high group exhibited low immune infiltration and activation. In other words, B7H4-high identifies immuno-cold tumors in CeCa. Moreover, the prognosis of these subgroups was consistent with immunogenicity, namely the B7H4-high group exhibited the shortest OS and PFS, which was defined as “cold” tumors.

There is increasing evidence exhibiting that the activation of several oncogenic pathways contributes to the non-inflamed T-cell phenotype and is responsible for the resistance to immunotherapy. Knockdown and pharmacological inhibition of PPARG could notably increase cytokine expression promote the sensitivity to immunotherapy [21]. In Wnt/β-catenin-positive melanoma tumors, decreased production of chemokine CCL4 leads to reduced recruitment of BATF3 DCs to the TME [22]. Besides, loss of PTEN activates the PI3K/AKT pathway, which is associated with an immuno-cold phenotype in melanoma [33]. In addition, the activation of oncogenic pathways also indicates the highly proliferative capacity of tumor cells. In this research, we found that B7H4 was positively correlated with the activities of PPARG and Wnt/β-catenin pathways and identifies highly proliferative subtypes. Moreover, consistent with the phenotype of “cold” tumors, B7H4-high predicts limited therapeutic potions in CeCa.

In recent years, potential biomarkers to distinguish “hot” and “cold” tumors have been studied extensively. Cai et al*.* reported that IFITM3 was upregulated in inflamed tumors and could be used as a feasible biomarker [19]. Hu et al*.* uncovered that SIGLEC15 shaped a non-inflamed tumor microenvironment in bladder cancer [34]. There is no doubt that these studies provide new perspectives to this field. In our research, we revealed the B7H4-PDL1 classifier was essential for clinical assessment to demarcate “hot” and “cold” in CeCa, which may be a useful biomarker for immunotherapy. However, the feasibility of these biomarkers was not tested using large-scale immunotherapy cohorts, which was the main shortcomings in our research and these studies. Thus, we believe that the B7H4-PDL1 classifier should undergo large-scale clinical assessment in immunotherapy cohorts before clinical application. Once its reliability could be confirmed, it will contribute to identifying the advantaged population who may benefit from immunotherapy.

## Conclusions

To sum up, we report that B7H4 is negatively correlated with PDL1 expression and immune cell infiltration. Besides, B7H4-high identifies immuno-cold tumors, highly proliferative tumors and predicts limited therapeutic potions in CeCa. Overall, the B7H4-PDL1 classifier is a convenient and feasible biomarker for the demarcation of tumor immunogenicity in CeCa.

## Supplementary Information

**Additional file 1:** Additional methods; **Table S1.** Table of abbreviations; **Table S2.** Detailed information of immunotherapy-related gene signatures.

**Additional file 2: Figure S1.** Correlations between B7H4 and immunological features in CeCa. (A) The correlation between PDL1 and T cell inflamed score in CeCa. (A) The correlation between B7H4 and T cell inflamed score in CeCa. (C) Correlations between B7H4 and common inhibitory immune checkpoints in CeCa. The color reveals the Pearson correlation coefficient.

**Additional file 3: Figure S2.** Correlations between B7H4-PDL1 classifier and immunological features in CeCa (supplement). (A) The proportion of B7H4-high, PDL1-high, co-low and co-high subgroups in CeCa. (B) Differences in the gene markers of the common TIICs in B7H4-high, PDL1-high and co-low subgroups. (C) Differences in T cell inflamed score in B7H4-high, PDL1-high and co-low subgroups.

## Data Availability

All data are included in the article, and data are available upon reasonable request.

## Abbreviations

ICB: Immune checkpoint blockade
PD1: Programmed cell death 1
PDL1: Programmed death-ligand 1
CeCa: Cervical cancer
TCGA: The Cancer Genome Atlas
CGGA: The Chinese Glioma Genome Atlas. TMA: tissue microarray
QIF: Quantitative immunofluorescence
IHC: Immunohistochemistry
IRS: Immunoreactivity score
OS: Overall survival
RFS: Relapse-free survival
IFN-γ: Interferon-γ
MHC-I: Major histocompatibility complex class I
