# Supplementary material for: The B7H4-PDL1 classifier stratifies immuno-phenotype in cervical cancer
Source: Cancer Cell Int. 2022 Jan 4;22:3. doi: 10.1186/s12935-021-02423-8 (PMC8728907; doi:10.1186/s12935-021-02423-8)
Supplement: Supplementary file 1 — Additional file 1: Additional methods; Table S1. Table of abbreviations; Table S2. Detailed information of immunotherapy-related gene signatures. [file 12935_2021_2423_MOESM1_ESM.docx]

**Supplementary Methods**

**Pan-cancer analysis of correlation between B7H4 and immunological features**

To assess the pan-cancer immunological correlation of B7H4, we first collected information on 122 immunomodulators including major histocompatibility complex (MHC), receptors, chemokines, and immunostimulators from the study of Charoentong *et al.* [1]. Then, the correlation between B7H4 and two major immune checkpoints PDL1 and CTLA4 was also assessed. Besides, TISIDB [2] tool was used to estimate the abundance of immune cells infiltration, and the correlation between B7H4 and immune cells infiltration was subsequently evaluated.

**Evaluation of the immunological features of the TME in CESC**

Immunological characteristics of the tumor microenvironment (TME) in CESC include the expression of immunomodulators, infiltration levels of tumor-infiltrating immune cells (TIICs) and activity of the cancer immunity cycle. First, ESTIMATE algorithm was performed to calculate the scores of tumor purity, the level of stromal cells, and the infiltration levels of immune cells in tumor tissues based on the transcriptome data of CESC [3]. Then, gene expression of 122 immunomodulators was included. Besides, in order to avoid calculation errors, we comprehensively calculated the infiltration level of TIICs using three independent algorithms: EPIC [4], ImmuneCell [5], and TISIDB [2]. Besides, we also collected the effector genes of TIICs, such as IL7R and NCR1 for natural killer (NK) cells, CD8A and IFNG for CD8+ T cells, SLC15A3 and IL21R for dendritic cells (DCs), MMP8 and MS4A6A for macrophages, and CTLA4 for Th1 cells.

Moreover, the cancer immunity cycle reflects the anti-cancer immune response and conceptualize as a series of stepwise events, including release of cancer cell antigens (Step 1), cancer antigen presentation (Step 2), priming and activation (Step 3), trafficking of immune cells to tumors (Step 4), infiltration of immune cells into tumors (Step 5), recognition of cancer cells by T cells (Step 6), and killing of cancer cells (Step 7). The activities of these steps above determine the fate of tumor cells, and the activation scores of each step can be calculated according to the expression of specific genes by utilizing single sample gene set enrichment analysis (ssGSEA) [6].

To confirm the role of B7H4-PDL1 classifier in assessing cancer immunity in CESC, we compared these immunological characteristics of TME between B7H4-high, PDL1-high and co-low groups with respect to the above aspects.

**Calculation of the enrichment scores of immunotherapy-related gene signatures**

According to previous research [7], we collected several gene-sets correlated with anti-tumor immunity such as genes involved in hypoxia and DNA replication. The enrichment scores of these signatures were calculated using the GSVA package [8]. Detailed information on these gene signatures was exhibited in Table S2.

**Prediction of therapeutic response**

The role of B7H4-PDL1 classifier in predicting the response to therapy was also evaluated. First, drug-target genes were screened using the Drugbank database (https://go.drugbank.com/). Nest, we also predicted the response to various therapies for each patient based on the Cancer Genome Project (CGP) database (<https://www.sciencedirect.com/topics/neuroscience/cancer-genome-project>). Several common therapeutic drugs. The prediction process was conducted by R package “pRRophetic” where the samples’ half-maximal inhibitory concentration (IC50) was estimated by ridge regression and the prediction accuracy was evaluated by 10-fold cross-validation based on the CGP training set. Default options were used for all parameters [9].

**Reference**

1. Charoentong P, Finotello F, Angelova M, Mayer C, Efremova M, Rieder D, et al. Pan-cancer Immunogenomic Analyses Reveal Genotype-Immunophenotype Relationships and Predictors of Response to Checkpoint Blockade. Cell Rep. 2017;18(1):248-62.

2. Ru B, Wong CN, Tong Y, Zhong JY, Zhong SSW, Wu WC, et al. TISIDB: an integrated repository portal for tumor-immune system interactions. Bioinformatics. 2019;35(20):4200-2.

3. Yoshihara K, Shahmoradgoli M, Martinez E, Vegesna R, Kim H, Torres-Garcia W, et al. Inferring tumour purity and stromal and immune cell admixture from expression data. Nat Commun. 2013;4:2612.

4. Racle J, de Jonge K, Baumgaertner P, Speiser DE, Gfeller D. Simultaneous enumeration of cancer and immune cell types from bulk tumor gene expression data. Elife. 2017;6.

5. Miao YR, Zhang Q, Lei Q, Luo M, Xie GY, Wang H, et al. ImmuCellAI: A Unique Method for Comprehensive T-Cell Subsets Abundance Prediction and its Application in Cancer Immunotherapy. Adv Sci (Weinh). 2020;7(7):1902880.

6. Xu L, Deng C, Pang B, Zhang X, Liu W, Liao G, et al. TIP: A Web Server for Resolving Tumor Immunophenotype Profiling. Cancer Res. 2018;78(23):6575-80.

7. Hu J, Yu A, Othmane B, Qiu D, Li H, Li C, et al. Siglec15 shapes a non-inflamed tumor microenvironment and predicts the molecular subtype in bladder cancer. Theranostics. 2021;11(7):3089-108.

8. Hanzelmann S, Castelo R, Guinney J. GSVA: gene set variation analysis for microarray and RNA-seq data. BMC Bioinformatics. 2013;14:7.

9. Geeleher P, Cox N, Huang RS. pRRophetic: an R package for prediction of clinical chemotherapeutic response from tumor gene expression levels. PLoS One. 2014;9(9):e107468.

**Table S1. Table of abbreviations.**

| **Abbreviation** | **Full name** |
| --- | --- |
| ACC | Adrenocortical carcinoma |
| BLCA | Bladder urothelial carcinoma |
| BRCA | Breast invasive carcinoma |
| CESC | Cervical squamous cell carcinoma and endocervical adenocarcinoma |
| CHOL | Cholangio carcinoma |
| COAD | Colon adenocarcinoma |
| DLBC | Lymphoid neoplasm diffuse large B-cell lymphoma |
| ESCA | Esophageal carcinoma |
| GBM | Glioblastoma multiforme |
| HNSC | Head and neck squamous cell carcinoma |
| KICH | Kidney chromophobe |
| KIRC | Kidney renal clear cell carcinoma |
| KIRP | Kidney renal papillary cell carcinoma |
| LAML | Acute myeloid leukemia |
| LGG | Brain lower grade glioma |
| LIHC | Liver hepatocellular carcinoma |
| LUAD | Lung adenocarcinoma |
| LUSC | Lung squamous cell carcinoma |
| MESO | Mesothelioma |
| OV | Ovarian serous cystadenocarcinoma |
| PAAD | Pancreatic adenocarcinoma |
| PCPG | Pheochromocytoma and paraganglioma |
| PRAD | Prostate adenocarcinoma |
| READ | Rectum adenocarcinoma |
| SARC | Sarcoma |
| SKCM | Skin cutaneous melanoma |
| STAD | Stomach adenocarcinoma |
| TGCT | Testicular germ cell tumors |
| THCA | Thyroid carcinoma |
| THYM | Thymoma |
| UCEC | Uterine corpus endometrial carcinoma |
| UCS | Uterine carcinosarcoma |
| UVM | Uveal melanoma |

**Table S2. Detailed information of immunotherapy-related gene signatures.**

| **Pathway** | **Reference** | **Genes** |
| --- | --- | --- |
| IFN-γ signature | PMID: 28650338 | TIGIT, CD27, CD8A, PDCD1LG2, LAG3, CD274, CXCR6, CMKLR1, NKG7, CCL5, PSMB10, IDO1, CXCL9, HLA-DQA1, CD276, STAT1, HLA-DRB1, HLA-E |
| APM signal | PMID: 31563503 | B2M, HLA-A, HLA-B, HLA-C, TAP1, TAP2 |
| DNA replication | PMID: 29443960 | DNA2, FEN1, LIG1, MCM2, MCM4, MCM6, MCM7, PCNA, POLA2, POLE, POLE2, PRIM1, PRIM2, RFC2, RFC3, RFC4, RFC5, RNASEH2A, RPA1, RPA3 |
| Nucleotide excision repair | PMID: 29443960 | CETN2, ERCC4, LIG1, PCNA, POLE, POLE2, RFC2, RFC3, RFC4, RFC5, RPA1, RPA3 |
| Proteasome | PMID: 29443960 | IFNG, PSMA4, PSMB2, PSMB4, PSMC4, PSMD4, PSMD7 |
| EGFR ligands | PMID: 31563503 | EGFR, AREG, AREGB, EREG, HBEGF, TGFA |
| FGFR3-coexpressed genes | PMID: 31563503 | FGFR3, TP63, IRS1, SEMA4B, PTPN13, TMPRSS4 |
| PPARG network | PMID: 27197067 | PPARG, IGFBP3, GDF15, MYH14, IHH, OCLN, AQP3, SCNN1G, PLIN5, KRT19, GPT, CYP4B1, UGT1A7, DGAT2, KRT20, SNCG, GSTA1, ACADL, BDH1, HMGCS2, LIPE |
| WNT/β-catenin network | PMID: 27197067 | CTNNB1, TFF1, HAPLN1, IHH, WNT7B, BMP7, SEMA5A, SCN5A, ERBB3, TSPAN8, EPCAM, TH, GPX2, GAD1, HSD17B2, KRT7, NOX1, CYB5A, CYP4F12, ID4, SIM2, MECOM, MSX2, KLF5, SMAD6, POU5F1, FOXQ1, GATA2, GATA3, EMX2 |
| Hypoxia | PMID: 31563503 | CAV1, COL5A1, ITGA5, P4HA2, SLC16A1, TGFBI, DPYSL2, SRPX, TRAM2, SYDE1, LRP1, PDLIM2, SAV1, AHNAK2, CAD, CYP1B1, DAAM1, DSC2, SLC2A3, FUT11, GLG1, GULP1, LDLR, THBS4 |
