# Supplementary figures and images for: The B7H4-PDL1 classifier stratifies immuno-phenotype in cervical cancer
Source: Cancer Cell Int. 2022 Jan 4;22:3. doi: 10.1186/s12935-021-02423-8 (PMC8728907; doi:10.1186/s12935-021-02423-8)

**A**

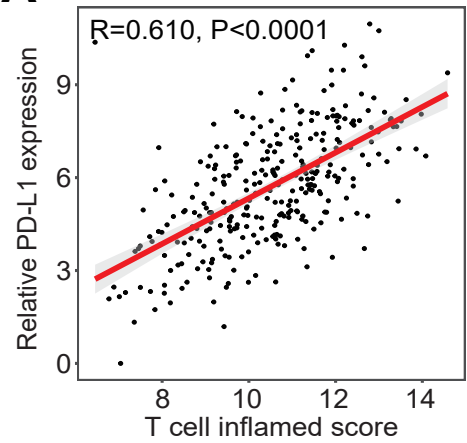

**B**

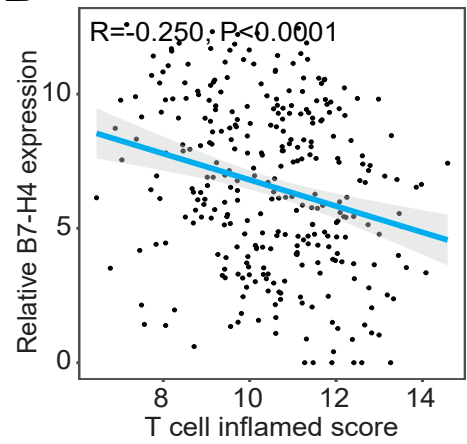

**C**

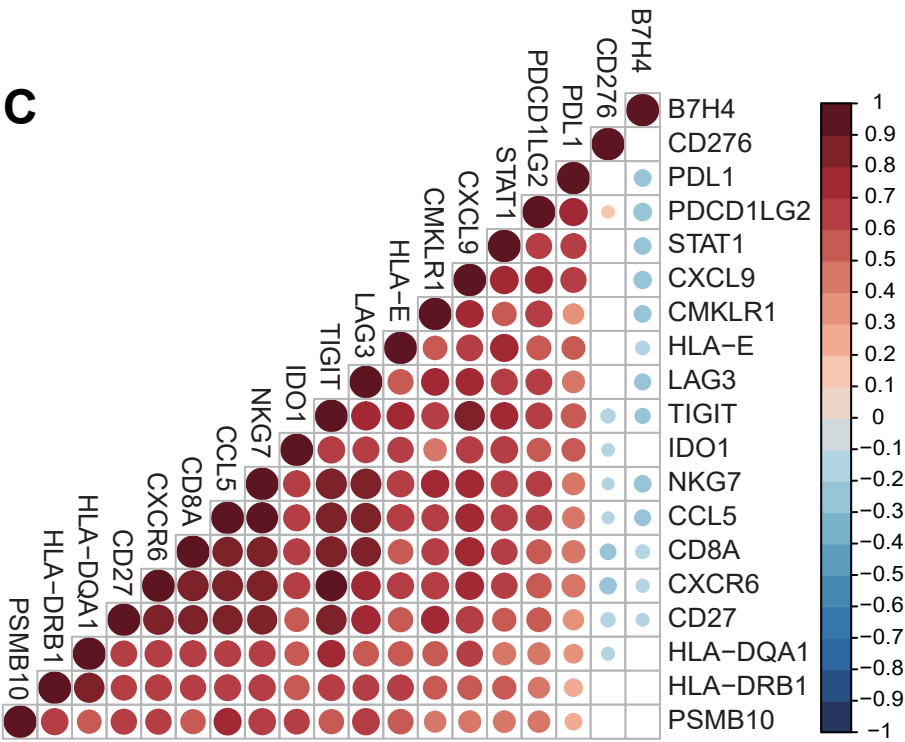

Supplement: Supplementary file 2 — Additional file 2: Figure S1. Correlations between B7H4 and immunological features in CeCa. (A) The correlation between PDL1 and T cell inflamed score in CeCa. (A) The correlation between B7H4 and T cell inflamed score in CeCa. (C) Correlations between B7H4 and common inhibitory immune checkpoints in CeCa. The color reveals the Pearson correlation coefficient. [file 12935_2021_2423_MOESM2_ESM.pdf]

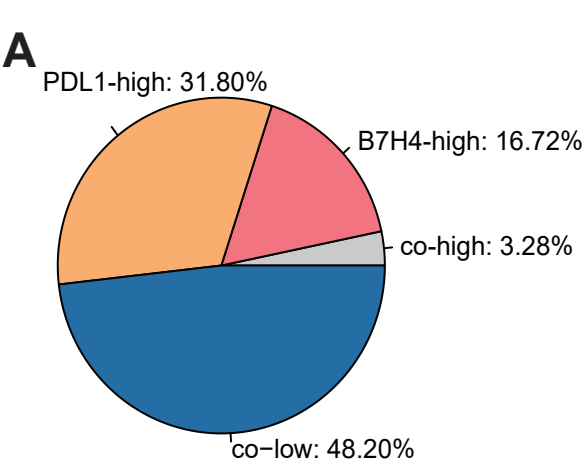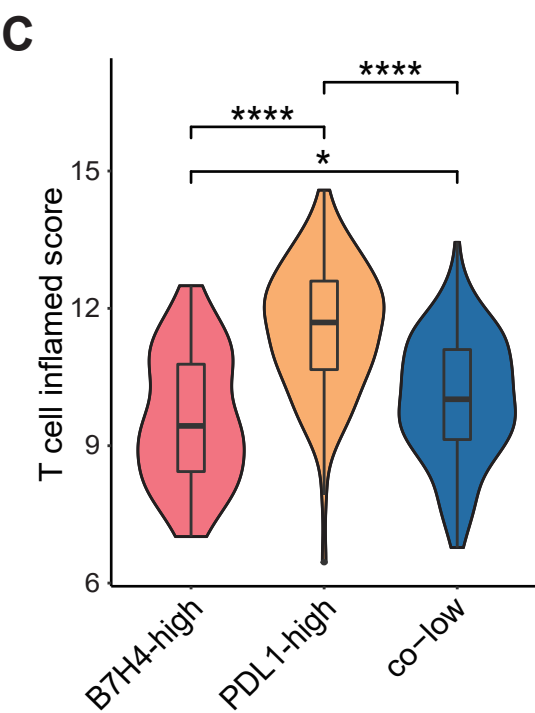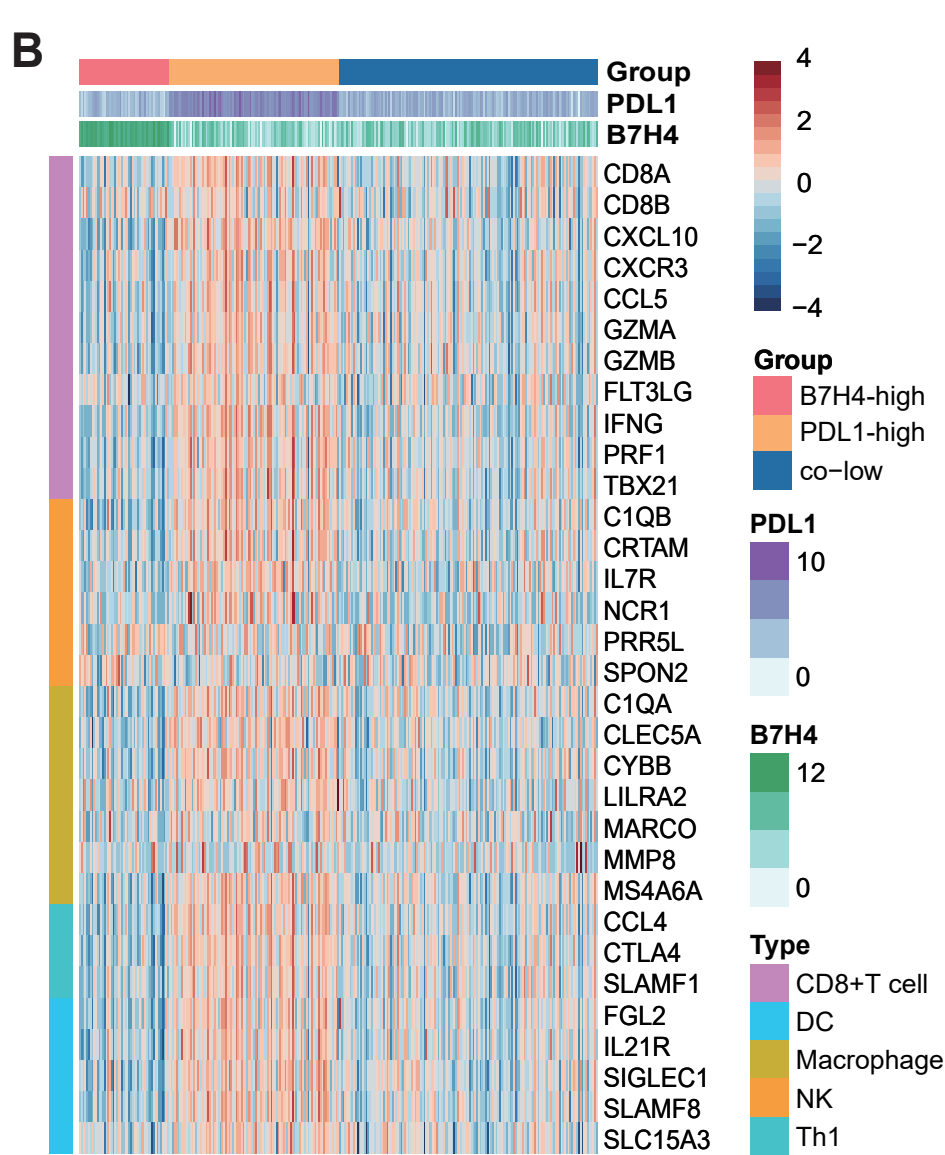

Supplement: Supplementary file 3 — Additional file 3: Figure S2. Correlations between B7H4-PDL1 classifier and immunological features in CeCa (supplement). (A) The proportion of B7H4-high, PDL1-high, co-low and co-high subgroups in CeCa. (B) Differences in the gene markers of the common TIICs in B7H4-high, PDL1-high and co-low subgroups. (C) Differences in T cell inflamed score in B7H4-high, PDL1-high and co-low subgroups. [file 12935_2021_2423_MOESM3_ESM.pdf]
